# Lettuce entertain you: Assessing *Sandwich Builder* as a measure of auditory short-term memory

**DOI:** 10.3758/s13428-025-02707-1

**Published:** 2025-06-04

**Authors:** Drew J. McLaughlin, Arthur G. Samuel

**Affiliations:** 1 Basque Center on Cognition, Brain and Language, Donostia-San Sebastián, Paseo Mikeletegi 69, 20009 Gipuzkoa, Spain; 2 Department of Psychology, Stony Brook University, Stony Brook, NY USA; 3 Ikerbasque, Bilbao, Spain

**Keywords:** Short-term memory, Auditory processing, Gamified research

## Abstract

Numerous language models propose a critical role of auditory short-term memory in spoken language processing, making reliable estimations of individual listeners’ memory capacities essential in linguistic research. Although a variety of cognitive tasks exist for assessing both visual and auditory short-term capacities in a laboratory environment (e.g., digit span), these types of tasks are typically not engaging for the participant. In the current study, we assess the validity and reliability of a novel “gamified” short-term memory assessment tool called *Sandwich Builder*. In *Sandwich Builder*, the participant completes 12 trials of a sandwich-building game. In each trial, a drive-thru customer’s sandwich order is presented auditorily, including a list of 1 to 10 sandwich ingredients (e.g., salami, cheddar cheese, mayo, and olives). The participant needs to remember these sandwich ingredients and then build the customer’s sandwich by selecting the necessary ingredients in the correct order. We evaluate *Sandwich Builder* as an estimate of auditory short-term memory, finding significant correlations with other classic short-term memory measures. Additionally, we measure affect (i.e., mood), fatigue, and motivation following *Sandwich Builder* as compared to classic tasks: Participants leave *Sandwich Builder* in a better mood, less fatigued, and more motivated than they leave other memory tasks. *Sandwich Builder* shows strong convergent, discriminant, and predictive validity, and moderate-to-good test–retest reliability. Finally, we examine the practicality of *Sandwich Builder* as a measure of individual differences in speech perception research, providing expected power curves for two common paradigms. Altogether, we robustly demonstrate the utility of *Sandwich Builder*, which is openly available online.

**Supplementary Information:**

The online version contains supplementary material available at 10.3758/s13428-025-02707-1.

## Lettuce entertain you: Assessing *Sandwich Builder* as a measure of auditory short-term memory

Estimating individual differences in memory abilities is critical in psycholinguistic research. Numerous language models propose a key role of auditory short-term memory in spoken language processing (e.g., the active control model; Nusbaum & Morin, 1992) and vocabulary acquisition (e.g., Gupta & MacWhinney, 1997), making reliable estimates of individual listeners’ memory capacities essential. Although a variety of tasks exist for assessing both visual and auditory short-term memory capacities in a laboratory environment (e.g., digit span), these types of experiments are often simultaneously effortful and not engaging for the participant—which may, in turn, negatively impact data quality (DeRight & Jorgensen, 2015). One increasingly popular approach in psychological science for circumventing this issue is experiment gamification (Lumsden et al., 2016). The aim of gamification is to incorporate features common to videogames (e.g., graphics, competition, and/or narrative) to convert what would typically be a boring experiment into something that will engross participants. Indeed, gamified experiments can increase participant motivation (Dörrenbächer et al., 2014) and performance (Ninaus et al., 2015). In the current study, we evaluate a novel gamified short-term memory assessment tool, *Sandwich Builder*, demonstrating its convergent, discriminant, and predictive validity, as well as test–retest reliability. We also investigate the benefits of gamification, comparing participants’ affect (i.e., mood), fatigue, and motivation following *Sandwich Builder* versus classic short-term memory tasks.

Memory researchers have distinguished long-term, short-term, and working memory (Cowan, 2008). Whereas long-term memory encompasses an extensive repository of knowledge and past experiences, short-term and working memory are proposed to have limited capacities and shorter decay rates. The distinction between short-term and working memory lies in the manipulation of held information—short-term memory refers to the cognitive system used for holding units of information before recall (e.g., digits or words), while working memory refers to the cognitive systems used for maintenance and manipulation before recall (Cowan et al., 2005). In the classic working memory model by Baddeley and Hitch (1974), three subsystems were proposed: the central executive, the phonological loop, and the visuospatial sketch pad. Although the exact nature of this working memory model has evolved (e.g., Baddeley, 2000), the general concept of a multi-feature system used to store and manipulate units remains. In practical research use, however, the distinction between short-term and working memory is often clouded: The same digit span tasks performed in forward (i.e., recalling 1, 2, 3 as 1, 2, 3) versus reverse (i.e., recalling 1, 2, 3 as 3, 2, 1) directions are typically referred to as a measures of short-term memory versus working memory, respectively—despite evidence to support this distinction remaining mixed (St Clair-Thompson, 2010). The current study will focus on confirming *Sandwich Builder* as a measure of auditory short-term memory, given the “forward recall” nature of the task’s design.

Several measures of short-term/working memory exist that are focused on estimating auditory capacities. Those most prominently used in psycholinguistic research as measures of individual differences are (1) auditory forward/backward digit span (adapted from the Wechsler Adult Intelligence Scale, see Wechsler, 1987), (2) listening span (Pichora-Fuller et al., 1995; adapted from reading span, see Daneman & Carpenter, 1980), and (3) the Word Auditory Recognition and Recall Measure (WARRM; Smith et al., 2016). In the auditory digit span task, participants are presented with a series of digits (e.g., 5-6-3) on a trial and instructed to recall them in either the same (forward) order or backwards (i.e., 3-6-5). Forward recall is typically interpreted as an index of short-term memory while backward recall is interpreted as an index of working memory, with list lengths indicative of capacity. The listening span task typically follows a similar design, but with presentations of words (and, in some cases, words presented in background noise; Pichora-Fuller et al., 1995). A variation of the listening span task is the “free recall” task, which presents many (e.g., 10, 20, or 30) words in rapid succession on each trial and then examines how serial position, length of list, and interstimulus interval impact the number of words recalled (Postman & Phillips, 1965). The WARRM combines listening span (in quiet) with an estimate of word recognition accuracy (i.e., confirming participants accurately perceive items); it is particularly well suited to audiology research in populations with hearing loss.

While the auditory digit span and free recall tasks can be easily ported to online data collection platforms, the latter, WARRM, is specifically designed to be administered in person by the researcher. All three tasks are typically considered effortful and repetitive. To the best of our knowledge, no tools currently exist that focus on measuring auditory short-term memory in an engaging manner. There are, however, several gamified visual working memory tasks. McPherson and Burns (2008) examined the validity of using two games, Space Matrix and Space Code, as estimates of working memory and/or processing speed. In Space Matrix, a dot matrix task (Miyake et al., 2001) is incorporated into a game in which the user is trying to fire lasers at enemy spaceships. The dot matrix task requires the participant to “add together” two visuals of interconnected dots (in a 3 × 3 grid). Space Matrix demonstrated convergent validity (i.e., significant correlations) with classic measures of processing speed, working memory, and fluid intelligence. One limitation of Space Matrix is that the game was not made available to other researchers; to the best of our knowledge, it has not been used in subsequent research studies.

Another example of a visual working memory task with gamification is Shapebuilder (Atkins et al., 2014). In Shapebuilder, participants are shown a 4 × 4 grid of various colored shapes and then must recall where each object goes in the grid (via a drag-and-drop response). In Atkins et al.’s (2014) validation study, Shapebuilder scores showed convergent validity with classic working memory tasks (e.g., *N*-back) and predictive validity for Raven’s Progressive Matrices (Raven, 2003). Notably, Shapebuilder has relatively few game-like aspects (e.g., no theme or story). In contrast, Ninaus et al. (2015) examined effects of multiple gamification elements (e.g., progress bar, level indicator, and thematic setting) on performance during a visual working memory task. Participants who completed the gamified version of the task outperformed participants who completed the classic version of the task.

### The current study

We assess a novel gamified experiment, *Sandwich Builder*, as a measure of auditory short-term memory. *Sandwich Builder* has been created with Gorilla, an online platform for experimental design (Anwyl-Irvine et al., 2020), and incorporates multiple gamification elements (e.g., theme, levels, graphics, and animations). In the task, the participant completes 12 trials of a sandwich-building game. On each trial, a drive-thru customer’s sandwich order is presented auditorily, including a list of 1 to 10 sandwich ingredients (e.g., salami, cheddar cheese, mayo, and olives). The participant needs to remember these sandwich ingredients and then build the customer’s sandwich by selecting the necessary ingredients in the correct order.

*Sandwich Builder* was developed to measure short-term memory in a way that participants find less aversive. We predicted that *Sandwich Builder* scores would positively correlate with classic measures of (auditory and visual) short-term memory, demonstrating convergent validity. We also expected that participants would report better affect (i.e., mood), less fatigue, and greater motivation after completing *Sandwich Builder* than classic short-term memory tasks, reflecting the benefits of gamification. By inviting participants to complete a second session of *Sandwich Builder* approximately 2 weeks later, we aimed to establish *Sandwich Builder*’s test–retest reliability.

We also sought to examine the predictive validity of *Sandwich Builder*, hypothesizing that *Sandwich Builder* scores would be related to performance on challenging speech perception tasks. To this aim, we incorporated two speech transcription tasks: The first task involved transcription of speech-in-noise stimuli taken from McLaughlin et al. (2021), which had found a relationship between accuracy and WARRM (i.e., auditory working memory) scores in older adults. The second task involved transcription of second-language (L2, or “foreign”)-accented stimuli taken from McLaughlin et al. (2023), which had found a relationship between accuracy and WARRM scores in younger adults. We also tested whether *Sandwich Builder* scores may interact with participant age—i.e., whether relationships between *Sandwich Builder* scores and performance may be stronger in older than in younger adult populations. We predicted an interaction would emerge for the speech-in-noise task, matching the trends found in McLaughlin et al. (2021) in older—but not younger—adults. McLaughlin et al. (2023) only examined younger adults, so the question of whether the same relationship between memory and performance would be found in an older population remained open.

Finally, we predicted that *Sandwich Builder* scores would show no relationship with a theoretically unrelated measure of personality, demonstrating discriminant validity. The composite measure of extraversion from the Big 5 Inventory (John & Srivastava, 1999) was selected for this purpose, given prior evidence showing no relationship with verbal or spatial working memory (Waris et al., 2018).

## Method

### Transparency and openness

This study complies with transparency and openness guidelines. The study design, hypotheses, and analysis plan were pre-registered with Open Science Framework (https://osf.io/yaujp). Data, analysis scripts, and materials for the experiment can be found at: https://osf.io/snmqt/files/osfstorage. The *Sandwich Builder* task is available on Gorilla’s Open Materials at: https://app.gorilla.sc/openmaterials/864906. The study was approved by the Ethics Committee at the Basque Center on Cognition, Brain and Language (BCBL).

### Participants

Adult (aged 18–65; 50% female, 50% male, < 1% non-binary) first-language English participants (*N = *256) were recruited to complete Session 1. Of this sample, *n = *209 returned for Session 2. The Session 1 exclusions included four participants who reported neurological impairments and one who reported an error with the task audio. Two additional participants flagged during outlier identification were removed from both datasets for having a difference in Session 1 and Session 2 *Sandwich Builder* scores greater than five^1^ mean absolute deviations from the mean (Fig. S1 of the Supplemental Materials). Thus, the corresponding sample size for Session 1 analyses (convergent validity tests and analyses of reported affect, motivation, and fatigue) was *N = *249, while the final sample size for across-session (reliability test) and Session 2 analyses (predictive and discriminant validity tests) was *n = *207. Participants were recruited from Prolific (Palan & Schitter, 2018) and completed the study online through the Gorilla platform (Anwyl-Irvine et al., 2020). Fluency in additional languages was allowed so long as English was reported as a language learned from birth and was the participant’s dominant language. Approximately 33% of the total sample reported some knowledge of a language in addition to English. Only 10% of the total sample learned a second language before or at age 5, and only 7% of the total sample learned a second language from birth (simultaneous with English). Selection criteria on the Prolific recruitment platform screened out participants with known auditory or neurological impairments, and only allowed participants born in, raised in, and currently living in the United States. Participants were compensated $11.71 (the equivalent of £9.00) per session and a $1.95 (£1.50) bonus if they returned for Session 2.

Sample size was determined from (correlation) power analyses conducted with the *pwr* package in *R* (Champely et al., 2018), focusing on power to detect effects in the convergent validity analyses. These analyses indicated that correlations as small as *r* = 0.20 would be detectable at 90% power with 250 participants.

### Materials

#### Sandwich Builder

The list of 21 sandwich ingredients for *Sandwich Builder*, and their respective drawings, can be viewed in Appendix A.^2^

Two first-language (L1) speakers of American English, one male and one female, recorded the auditory stimuli for the *Sandwich Builder* task. Stimuli were first intensity-normalized (to 65 dB) in Praat (Boersma & Weenink, 2024). Next, to create the illusion of numerous customers passing through the drive-thru, each of these voices was also pitched up and pitched down (i.e., creating a total of six voices, three unique pitches per sex). Pitch manipulation was completed in Adobe Audition (Build 23.5.0.48) with the *Stretch and Pitch (process)* effect. Within the effect pane, the *Lock Stretch and Pitch Shift (Resample)* feature was selected to maintain typical human vocal qualities (i.e., preventing unwanted “slow-mo” and “chipmunk” voice effects).

The carrier phrase (*“I’d like a sandwich with…”*), sandwich ingredients (e.g., *“mayo”*), and conjunction (*“and”*) were all recorded in isolation. This allowed for a virtually unlimited number of combinations when creating stimuli in which customers placed a sandwich order. Random sandwich ingredient combinations for each level of *Sandwich Builder* were generated with a custom R script, and audio files were combined with a custom Python script (both available for re-use and further customization). Five-hundred milliseconds of silence was inserted between each audio file during combination. The conjunction *“and”* always occurred before the last item in the list. Altogether, this process created combined files such as *“I’d like a sandwich with arugula, mayo, pickles, and salami.”*

Visual materials for *Sandwich Builder* were custom-drawn using the Procreate app (version 5.3.10) on an iPad Air (OS version 17.5.1). In-experiment text used the fonts BFC Science Teacher, BFC Magic Marker, and BFC Spark (purchased from Blush Font Co., 2023). To reduce file sizes (and corresponding experiment loading times), images were compressed to a smaller size that maintained visual quality (compressed with iLoveIMG, n.d.).

#### Digit span

The same male and female L1 speakers of (American) English recorded the auditory digit span stimuli. Digits from 0 to 9 were recorded in isolation, intensity-normalized (to 65 dB) in Praat (Boersma & Weenink, 2024), and then matched for duration (to 352 ms, the average of all original files). Duration manipulation was completed with the *Stretch and Pitch (process)* effect in Adobe Audition (Build 23.5.0.48). Within the effect pane, the *Preserve Speech Characteristics* feature was selected to maintain unique talker vocal qualities (i.e., preventing changes to voice pitch). No pitch-shifted versions were created for this task.

Random series of digits (between 0 and 9) for each span level were generated with a custom R script, and then audio files were combined with a custom Python script. The male and female speakers’ voices were not intermixed within a sequence; half of the auditory trials were sequences of the male speaker only and the other half of the female speaker only. Sequences did not contain any repeated numbers. Unique sequences were generated for the auditory and visual versions of the task. Five hundred milliseconds of silence was inserted between each audio file during combination, as in the *Sandwich Builder* stimuli. For the visual version of the task, digits appeared onscreen for 352 ms with 500 ms, interstimulus intervals of blank screen—thus matching the timing of the auditory version of the task. No conjunction (*“and”*) was included within the series of digits presented in either version of the task.

#### Free recall

High-frequency, two-syllable nouns were selected for the free recall task^3^ using the MRC Psycholinguistic Database (version 2.00; Wilson, 1988). The same male and female L1 speakers of (American) English who recorded the *Sandwich Builder* and digit span stimuli also recorded the 60 words for free recall. Words were recorded in isolation and intensity-normalized (to 65 dB) in Praat (Boersma & Weenink, 2024).

Audio files were combined into six pseudorandom 10-word sequences with a custom Python script. The same pseudorandom combinations were used for all participants. Five hundred milliseconds of silence was inserted between each audio file during combination, as in the *Sandwich Builder* and digit span stimuli. The male and female speakers’ voices were not intermixed within a sequence; three of the auditory trials were sequences of the male speaker only and the other three of the female speaker only. For the visual version of the free recall task, timing matched the visual digit span task: Words appeared onscreen for 352 ms, with 500 ms interstimulus intervals of blank screen. No conjunction (*“and”*) was included for either version of the task.

#### Sentence-in-noise transcription

Materials for the sentence-in-noise transcription task included a subset of 16 stimuli taken from McLaughlin et al. (2021), which found a relationship between auditory working memory capacity (measured with the WARRM; Smith et al., 2016) and transcription accuracy in older adults. The recordings created for that study were of a female L1 (American) English talker reading sentences developed by Van Engen et al. (2012), which contain four key words each (e.g., “the **gray mouse ate** the **cheese**”). This L1 talker was not heard in any other tasks in the present study. The long-term average spectrum of the files was computed with Praat to generate speech-shaped noise (version 6.0.16; Boersma & Weenink, 2024). Sentences were then mixed with noise at a signal-to-noise ratio (SNR) of −4 dB. In McLaughlin et al. (2021), the −4 dB SNR resulted in average transcription accuracy of 66% in older adults and 91% in younger adults.

#### Second-language (L2) accent transcription

Materials for the L2 accent transcription task included a subset of 24 stimuli taken from McLaughlin et al. (2023), which found a relationship between auditory working memory capacity (measured with the WARRM; Smith et al., 2016) and transcription accuracy in young adults. Recordings were of three female Mandarin Chinese-accented speakers of English (i.e., whose L1 is Mandarin Chinese and L2 is English) producing semantically unpredictable sentences (Nye & Gaitenby, 1974) with four keywords each (e.g., *“the wrong shot led the farm”*). Sentences (8 per speaker) were presented in quiet. Transcription accuracy in McLaughlin et al. (2023) was approximately 60%.

#### Post-task questionnaire

After each of the five memory tasks, participants completed a questionnaire assessing affect (mood/emotional state), fatigue, and motivation. A full list of the items for each composite measure is provided in Appendix B.

Questions for the affect portion of the assessment were based on Dragojevic and Giles (2016). These items included questions in the frame: *“How ___ are you feeling?”* Negative valence prompts included irritated, annoyed, and frustrated, and positive valence prompts included interested, enthusiastic, and happy. Questions were presented visually in the same pseudorandom order for all participants. Responses were collected with a slider bar (mapping onto values from 0 to 100) with the leftmost (0) endpoint labeled *“not at all”* and the rightmost (100) endpoint labeled *“extremely.”*

#### Extraversion questionnaire

A subset of eight questions pertaining to extraversion from the Big Five Inventory questionnaire (items 1, 6, 11, 16, 21, 26, 31, and 36; John & Srivastava, 1999) was presented visually. The format of these questions was always *“I see myself as someone who…”* followed by the prompt (e.g., *“is talkative”*). Three of these eight items were reverse-scored, as in the original questionnaire. Participants responded with a five-point Likert scale from 1 (disagree strongly) to 5 (agree strongly). In the middle of the extraversion questionnaire, one attention check question was inserted (*“I see myself as someone who… is paying attention pick 5”*). Questions were presented in the same pseudorandom order for all participants.

#### Other questionnaires

Additional questionnaire items were included at the end of Session 1 to determine participants’ demographic information (age, gender, etc.). A set of questions from the Hearing Handicap Inventory for the Elderly (HHIE; Ventry & Weinstein, 1982) was included to assess potential hearing loss in the older adults in the sample. However, as this measure did not account for significant variance in any of the analyses, we do not discuss it further.

### Procedures

Participants completed the Session 1 tasks (*Sandwich Builder*, digit span [auditory], digit span [visual], free recall [auditory], and free recall [visual]) in one of five possible counterbalanced orders (i.e., a Latin square). Each task was followed by the post-task questionnaire, assessing affect (mood), fatigue, and motivation. Session 2 was completed in a set order: (1) *Sandwich Builder*, (2) speech-in-noise transcription, (3) accent transcription, and (4) the extraversion subset of the Big 5 Inventory questionnaire. Sessions 1 and 2 lasted approximately 50 and 30 min, respectively. All tasks were created with Gorilla software. Participants were instructed that there was no benefit to cheating (i.e., writing down answers on paper) during the tasks. Specifically, they were told that poor performance would not impact their payment, and were encouraged to perform without cheating to ensure the validity of the study’s data.

#### Sandwich Builder

**Fig. 1:**
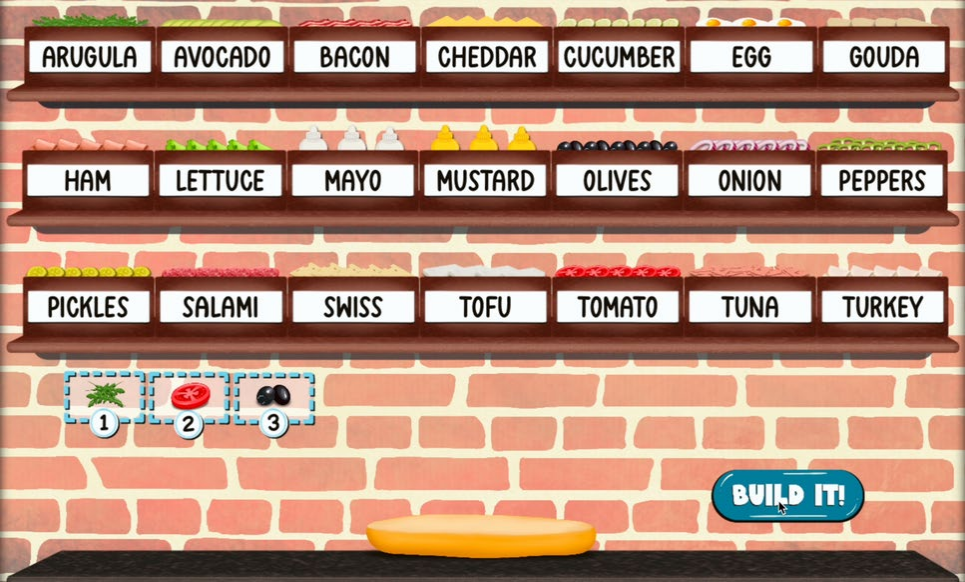
The assembly scene of *Sandwich Builder* is shown for a Level 3 trial. This example shows the scene after the participant has chosen three ingredients (arugula, tomato, and olives)

*Sandwich Builder* includes one practice trial and 12 test trials, lasting approximately 9.5 min. On each trial, a drive-thru customer’s sandwich order is first presented auditorily, including a list of 1 to 10 sandwich ingredients (e.g., salami, cheddar cheese, mayo, and olives). The current level (e.g., Level 2), corresponding to the number of sandwich ingredients, is shown to the participant before presentation of the auditory stimulus. The participant needs to remember the presented sandwich ingredients, in order, and then build the customer’s sandwich by selecting the necessary ingredients with mouse clicks. Figure 1 shows the sandwich assembly scene through which responses are collected. Following a click on a sandwich ingredient, an animation of that ingredient moving down to a “staging area” plays. The staging area shows the participant their selections in numbered order. Sandwich ingredients can only be selected one time, and selections cannot be deleted from the staging area. Once the participant has selected their ingredients, they submit their response by clicking an onscreen button that says: *“BUILD IT!”* Participants can submit their response after selecting any number of ingredients; for example, on Level 5 the participant ought to recall five ingredients, but the program will allow the participant to submit their response with only four (or fewer) ingredients in the staging area. It is not possible to select more ingredients than there are spots in the staging area (i.e., Level 5 has a maximum of five slots). After the participant submits their response, an animation of the sandwich being wrapped and delivered to the drive-thru window plays. A video demo showing the participant’s point of view when completing a trial of *Sandwich Builder* is provided in the Supplemental Materials.

**Fig. 2:**
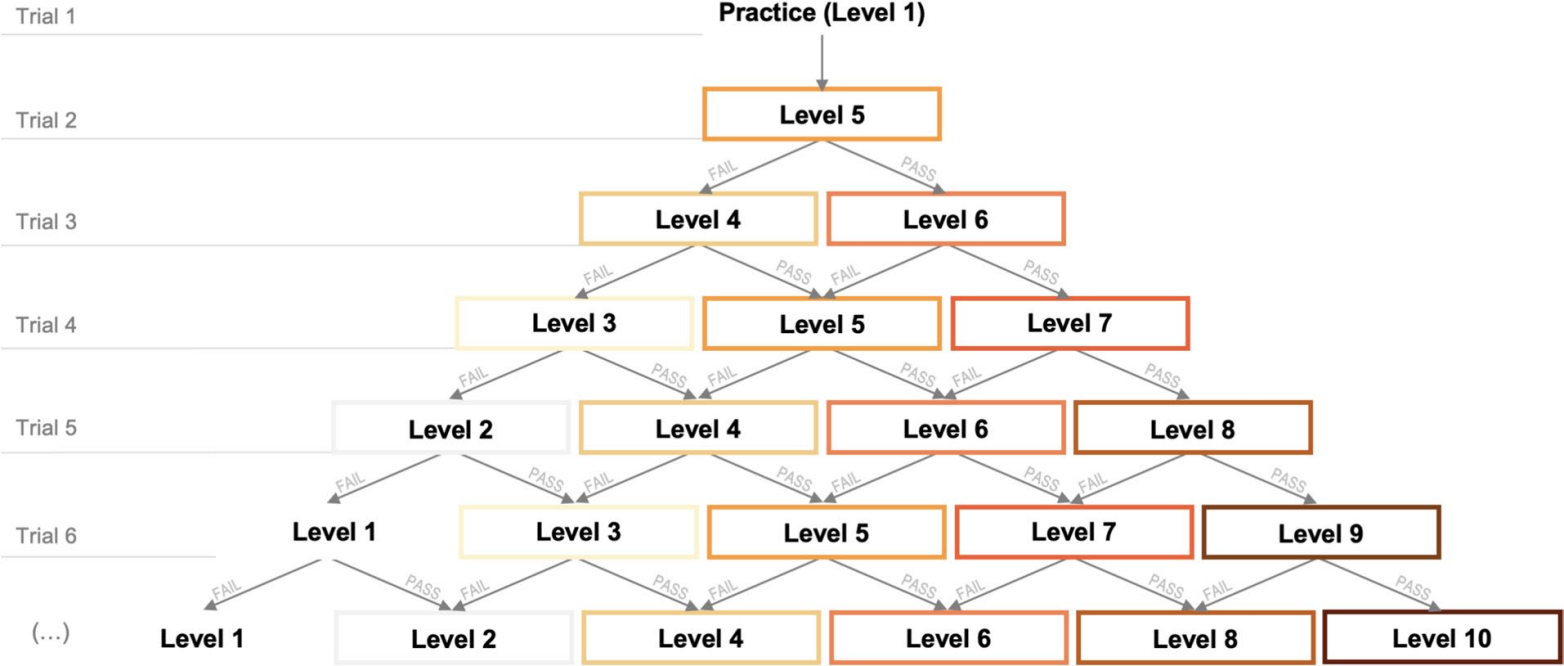
The trial flow for the first seven trials of *Sandwich Builder* is shown. The task begins with a Level 1 (i.e., one ingredient) practice trial, then presents the test trials starting from Level 5 (i.e., five ingredients). The difficulty of the subsequent trial depends on whether the participant passes or fails the current trial. A total of one practice and 12 test trials are presented

The practice trial for *Sandwich Builder* is a Level 1 (i.e., single ingredient) sandwich order. Guided instructions are presented during this trial that point the participant to where they should click. After the practice trial, participants begin the test trials, starting with a Level 5 sandwich order (Fig. 2).^4^ When the participant gets an order correct—meaning all ingredients are clicked on in the correct order—they are moved up a level in difficulty. When the participant fails—either choosing one or more incorrect ingredients, or selecting them in the wrong order—they are moved down a level in difficulty. The process continues until all 12 test trials are complete.

#### Digit span

Both the auditory and visual versions of the digit span task presented 36 trials in total (four per level). Half of the items per level were produced by the male talker and half by the female talker. Levels were presented in ascending order (from a span of 2 to a span of 10); within a level, items were presented in random order. For the auditory version, the audio was presented while a fixation cross was shown onscreen, and for the visual version, digits appeared for durations of 352 ms (matching the durations of the auditory digits), with 500 ms interstimulus intervals of blank screen. After the presentation of each sequence, participants typed the numbers they heard/saw into a response box. Instructions emphasized that participants must type the numbers in the correct order, and that spaces and punctuation were unnecessary.

#### Free recall

The free recall task was modeled after Smith et al. (2016). Unlike the digit span task, the free recall task contained only six trials. Each trial presented 10 words in rapid succession and instructed the participant to type out as many of the words as they could remember into a response box (separated by spaces) after the last word was presented. Instructions encouraged participants to type the word sequence in the correct order, though scoring procedures credited disordered sequences. The visual version of the task matched the design of the visual digit span: Words appeared for durations of 352 ms, with 500 ms interstimulus intervals of blank screen. The auditory version did not duration-match the items, but did space items with a 500 ms interstimulus interval.

#### Transcription tasks

The sentence-in-noise transcription task (semantically normal sentences such as *“the gray mouse ate the cheese”* presented in speech-shaped noise) and the L2 accent transcription task (semantically anomalous sentences such as *“the wrong shot led the farm”* presented in quiet) presented 16 and 24 test trials, respectively; each also contained one catch trial (“I am paying attention” spoken clearly by an L1 English speaker without background noise). Both tasks presented audio while a fixation cross was onscreen. After auditory presentation finished, a response box appeared for the participant to type what they heard. In both transcription tasks, stimuli were presented in random order.

### Analysis

#### Sandwich Builder scoring procedures

The accuracy of each individual trial was scored automatically during testing (i.e., within Gorilla to determine whether the participant passed/failed a trial and should move up/down in difficulty for the next trial). All sandwich ingredients had to be selected in the correct order to pass a trial. A participant’s final score was computed by summing a “base” score and a “partial credit” score. The base score for each participant was equal to the highest level passed at least two times. For example, if a participant passed Level 5 twice and Level 6 twice (and passed no levels above 6), their base score would be 6.0. The number of passes above that base-score level was then tallied for partial credit. If a participant passed Level 6 twice (and 6.0 was therefore the base score) but also passed Level 7 once, the above-base-score tally would be equal to 1. This tally was multiplied by 0.5 for the partial credit points. For example, a participant who passed Level 4 twice, Level 5 three times, Level 6 twice, and Level 7 once (and passed no other levels) would have a base score of 6.0 and a partial credit score of 0.5, equal to a final score of 6.5.^5^

Using this scoring system, the majority of participants (56%) in our Session 1 sample did not receive any partial credit—i.e., they did not pass any levels higher than their base-score level. The next most frequent partial credit score was 0.5 (37% of participants). Only a small proportion of participants (7%) had a more distributed score range that resulted in partial credit greater than 0.5. For example, one participant had a partial credit score of 1.5 because they passed Level 5 three times, Level 6 twice (base-score level), Level 7 once, Level 8 once, and Level 9 once. It is possible that participants with more distributed scores, such as in this example, develop a more effective strategy partway through the task, thereby improving their performance. As noted, this did not occur often.

To facilitate future use, we have created an R package, *orderup*, with a function for automatically scoring *Sandwich Builder* data following the procedure outlined above. The package can be installed directly from GitHub at: https://github.com/mclaughlindrew/orderup/. Examples of how to install and use the scoring function are provided.

#### Other task scoring procedures

For the auditory and visual digit span tasks, typed responses were compared against target number strings using a custom script (after removing spaces and any punctuation). A trial was scored as correct only if the typed number string was a perfect match to the target number string. The span score was calculated following the “mean span” rule reported by Woods et al. (2011), which is recommended over the original Wechsler (1987) scoring rule because it provides greater precision. First, the proportion of correct trials per list length is calculated (i.e., values of 0.00, 0.25, 0.50, 0.75, or 1.00). Next, the sum of proportions is added to a baseline value of 1.00 (i.e., 1.00 less than the initial tested list length, 2.00). Thus, a participant who passes all trials at list length 2 will obtain a span score of 2.00 (assuming no later trials are passed). Span scores can range from 1.00 to 10.00.

For the auditory and visual free recall tasks, typed responses were examined to determine how many of the target words were recalled each trial. Word order did not matter for the scoring of the free recall tasks (matching Smith et al., 2016). Each trial contained 10 target words and could thus award the participant up to 10 points. The average of scores (across the six total trials) was taken as the span score.

For the speech-in-noise and accent transcription tasks, trials were scored to determine the number of correctly identified keywords versus the number of incorrectly identified (or “missed”) keywords per sentence. The R package *autoscore* (Borrie et al., 2019) was used for automatic scoring. The following scoring features were used (i.e., set to TRUE): tense rule (scores differences such as “swipe” vs. “swiped” as correct), plural rule (scores differences such as “apple” vs. “apples” as correct), double letter rule (scores differences such as “leter” vs. “letter” as correct), and number text rule (scores differences such as “1” vs. “one” as correct). Common acceptable misspellings were also allowed; the list of misspellings comes partly from the autoscore package (items such as “absent” versus “abcent”) and partly from manual inspection of the data (items such as “gray” vs. “grey”).

#### Predictive validity model specifications

Generalized linear mixed-effects regression was used to model the recognition accuracy data from the speech-in-noise and accent transcription tasks in R (version 4.0.4; R Core Team, 2021) with the *glmer()* function from the *lme4* package (Bates et al., 2015). Likelihood ratio tests were conducted to determine the significance of effects of interest, and *p* values for model parameters were estimated using the *lmerTest* package (Kuznetsova et al., 2017). Recognition accuracy was treated as a grouped binomial, meaning that models predicted performance using two columns of data (number of correct words, number of incorrect/missed words) for each sentence. A logit link function was specified. Fixed effects included *Sandwich Builder* score and age, as well as the interaction between *Sandwich Builder* score and age^6^. Random intercepts were included by item and by subject. Model syntax is provided in Appendix C.

#### Affect, fatigue, and motivation model specifications

Linear mixed-effects regression in R (version 4.0.4; R Core Team, 2021) was used to separately examine ratings of affect, fatigue, and motivation following each of Session 1’s short-term memory tasks. Each of the composites of affect, fatigue, and motivation were centered and scaled prior to modeling. Fixed effects in all models included task followed (dummy-coded reference level: Sandwich Builder) and counterbalance (dummy-coded reference level: Order1), each containing five levels. Random effects included random intercepts by participant. Likelihood ratio tests were conducted to determine the significance of effects of interest, and *p* values for model parameters were estimated using the *lmerTest* package (Kuznetsova et al., 2017).

## Results

### Convergent validity

**Fig. 3:**
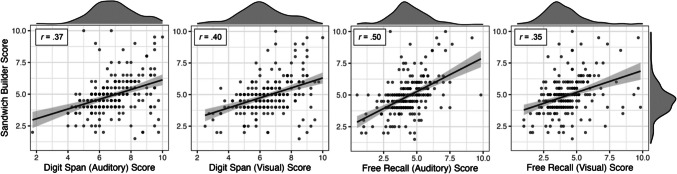
Correlations between *Sandwich Builder* scores and (from left to right) auditory digit span, visual digit span, auditory free recall, and visual free recall are visualized. Points represent individual participants. Density distributions of each task are shown to the right (*Sandwich Builder* scores) and above respective axes

Convergent validity was determined via correlations among the short-term memory tasks (Table 1, Fig. 3). All correlations were significant (Bonferroni-corrected *p < *.001). *Sandwich Builder* scores showed the strongest relationship with scores on the auditory free recall task (*r* = 0.50) followed by the visual digit span (*r = *0.40), auditory digit span (*r = *0.37), and visual free recall (*r = *0.35) tasks.

**Table 1:** Correlation matrix of short-term memory tasks

	*M*	*SD*	1. SB	2. DSA	3. DSV	4. FRA	5. FRV
1. *Sandwich Builder*	4.91	1.42	–				
2. Digit span (auditory)	6.81	1.37	0.37***	–			
3. Digit span (visual)	6.43	1.46	0.40***	0.65***	–		
4. Free recall (auditory)	4.33	1.34	0.50***	0.46***	0.62***	–	
5. Free recall (visual)	4.23	1.44	0.35***	0.53***	0.64***	0.66***	–

*** p < .001 (after Bonferroni correction); SB = *Sandwich Builder*; DSA = digit span (auditory); DSV = digit span (visual); FRA = free recall (auditory); FRV = free recall (visual)

#### Affect, fatigue, and motivation

**Fig. 4:**
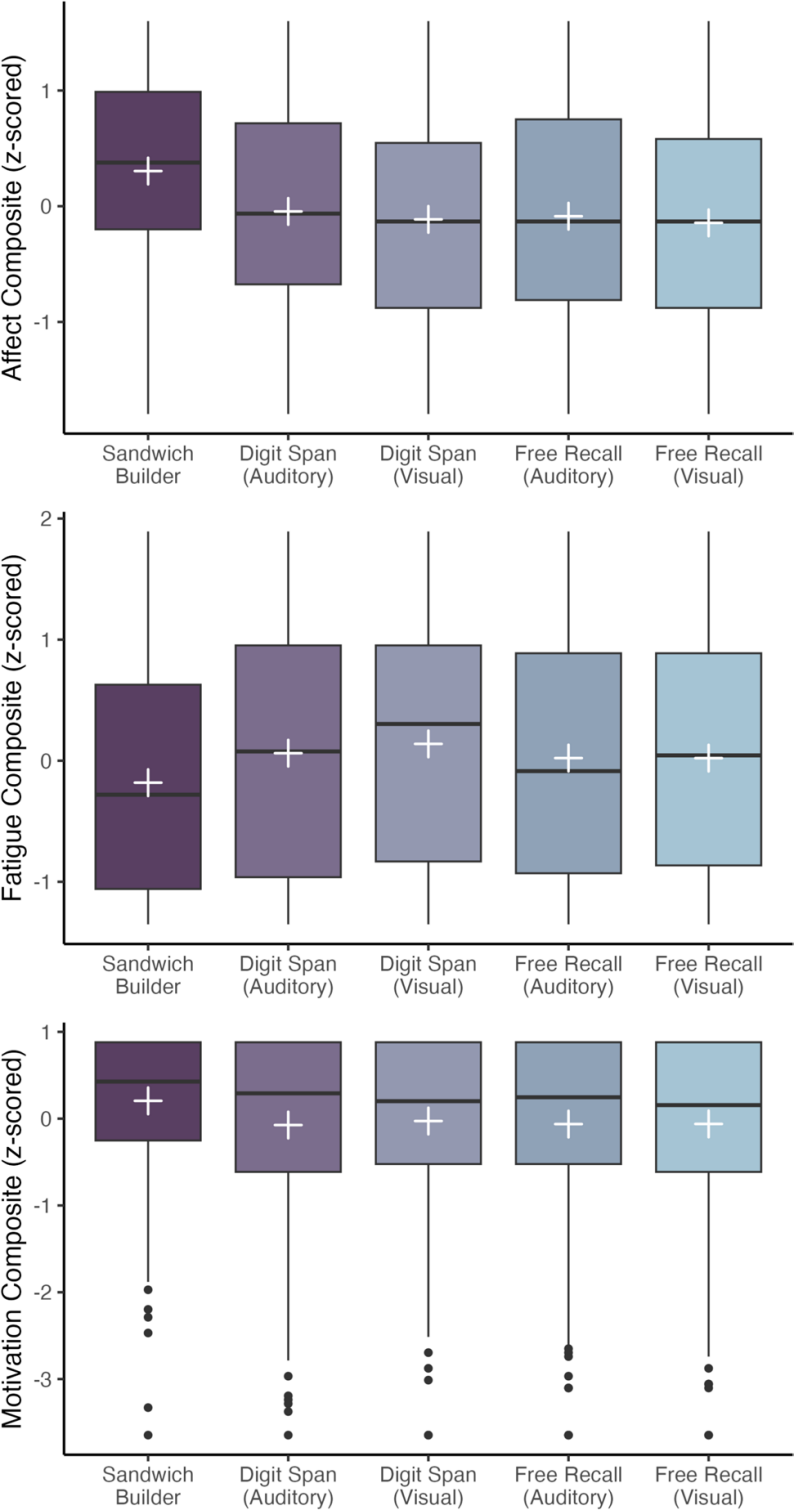
Ratings of affect (i.e., mood), fatigue, and motivation following each of the five short-term memory tasks are visualized. Boxplots show median (central line), interquartile range (box limits), and full data range (whiskers and outlier points). Overlaid white crosses show the means. Larger affect values correspond to more positive post-task mood (i.e., happier), larger fatigue values correspond to greater post-task tiredness, and larger motivation values correspond to greater desire to perform well at the task. All data were *z*-scored before creating composites

*Sandwich Builder* was created to see whether it is possible to measure short-term memory in a way that participants find less aversive. In fact, they reported more positive affect, less fatigue, and higher motivation following the *Sandwich Builder* task than following the other short-term memory tasks (Fig. 4). When directly asked which short-term memory task they would prefer to complete (if they had to do one of the five again), 79% of the Session 1 sample chose *Sandwich Builder*.

In the model predicting affect (i.e., mood) ratings, we examined the effect of task followed (*χ*^2^ = 122.58, *df = *4, *p < *.001) while accounting for any potential effect of counterbalance (*χ*^2^ = 6.49, *df = *4, *p = *.17). Model estimates indicated significantly more positive affect following *Sandwich Builder* than auditory digit span (*ß* = −0.35, *p < *.001), visual digit span (*ß* = −0.42, *p < *.001), auditory free recall (*ß* = −0.39, *p < *.001), and visual free recall (*ß* = −0.45, *p < *.001).

Using the same model structure to predict fatigue ratings, we again found an effect of task followed (*χ*^2^ = 40.76, *df = *4, *p < *.001) while accounting for any potential effect of counterbalance (*χ*^2^ = 4.28, *df = *4, *p = *.37). Model estimates indicated significantly less fatigue following *Sandwich Builder* than auditory digit span (*ß* = 0.24, *p < *.001), visual digit span (*ß* = 0.32, *p < *.001), auditory free recall (*ß* = 0.20, *p < *.001), and visual free recall (*ß* = 0.20, *p < *.001).

Finally, we examined motivation ratings, finding an effect of task followed (*χ*^2^ = 41.47, *df = *4, *p < *.001) while accounting for any potential effect of counterbalance (*χ*^2^ = 4.28, *df = *4, *p = *.37). Model estimates indicated significantly greater motivation following *Sandwich Builder* than auditory digit span (*ß* = −0.28, *p < *.001), visual digit span (*ß* = −0.23, *p < *.001), auditory free recall (*ß* = −0.27, *p < *.001), and visual free recall (*ß* = −0.27, *p < *.001).

### Reliability

**Fig. 5:**
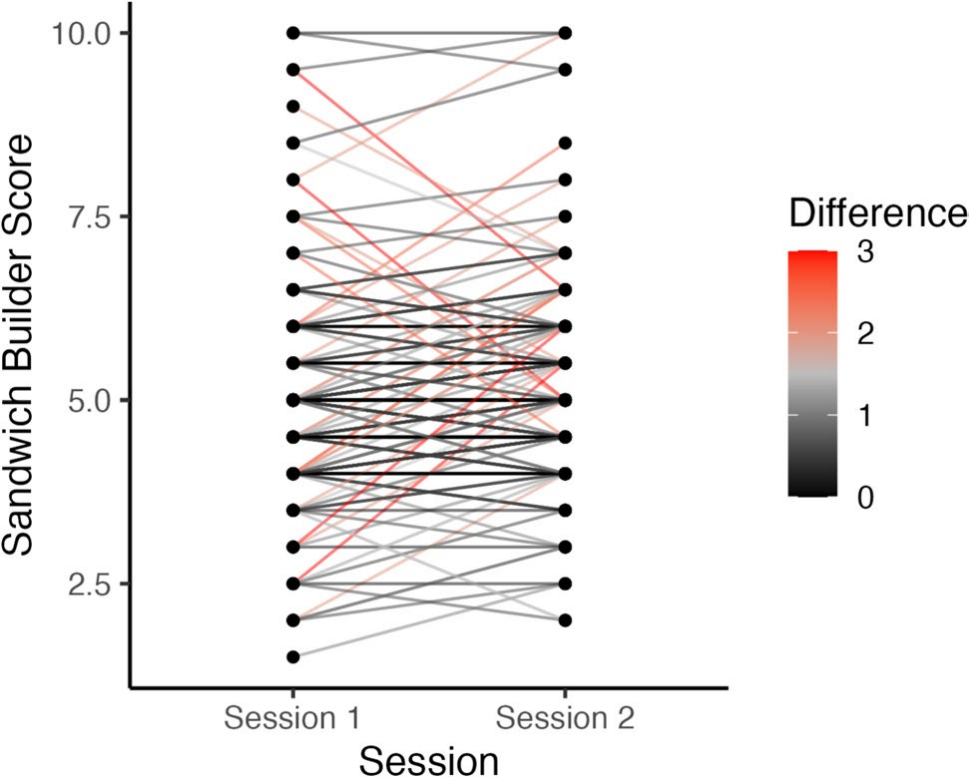
*Sandwich Builder* scores from Session 1 and Session 2 are shown with lines connecting individual subject points. A continuous scale from black (no change) to red (three-point score change) shows absolute difference in scores between sessions

Test–retest reliability was determined by comparing *Sandwich Builder* scores from Session 1 and Session 2 (Fig. 5). Results of a two-way random effects model of intraclass correlation (ICC), calculated with the *psych* package (Revelle & Revelle, 2015) in R, showed moderate-to-good reliability (ICC* = *0.75, 95% CI = [0.67–0.81])*.* As a rule of thumb, values from 0.50 to 0.75 are considered moderate and those from 0.75 to 0.90 are considered good (Koo & Li, 2016).

### Predictive validity

Predictive validity was determined by examining how *Sandwich Builder* scores predicted performance in two speech transcription tasks. Both of these speech-in-noise and second-language-accent transcription tasks had shown relationships with auditory working memory scores previously (McLaughlin et al., 2021; McLaughlin et al., 2023).

**Fig. 6:**
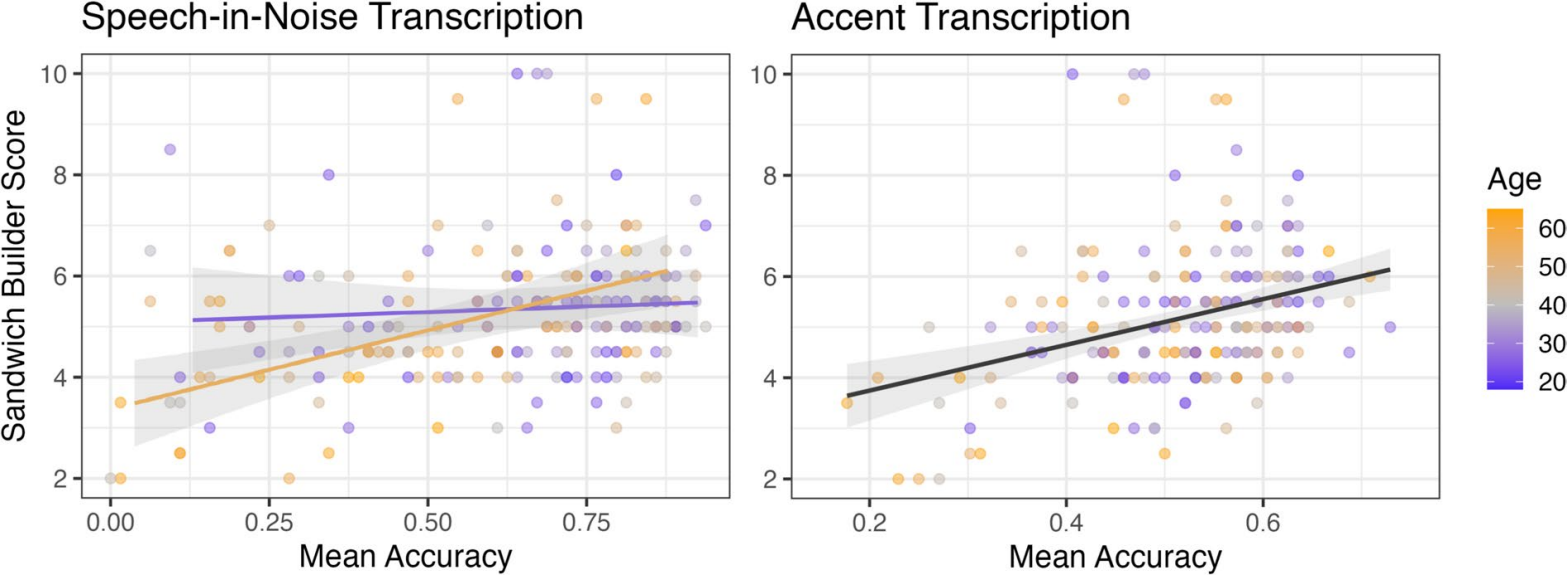
Performance on the speech-in-noise (left) and accent (right) transcription tasks is visualized as a function of *Sandwich Builder* scores and age. Points represent individual participants of ages corresponding to the legend’s color scale (i.e., 18 = purple, 65 = orange). In the speech-in-noise transcription plot, model fits of the bottom (18–30 years, purple) versus top (52–65 years, orange) quartiles of ages are shown with lines and standard error ribbons. Age was treated as a continuous variable in all models—fits of the bottom and top quartile are shown for visualization purposes only. In the accent transcription plot, a single fit line representing the trend for participants of all ages is shown. The interaction of *Sandwich Builder* scores and age was only significant in the speech-in-noise transcription task

In the speech-in-noise transcription task, log-likelihood model comparisons indicated that *Sandwich Builder* scores significantly improved model fit (*χ*^2^ = 15.18, *df = *1, *p < *.001). Before entering the interaction of *Sandwich Builder* scores and age into the model, the estimate for *Sandwich Builder* scores indicated an overall positive relationship with transcription accuracy (*ß* = 0.26, *p < *.001); in other words, participants with larger short-term memory capacities transcribed speech-in-noise with better accuracy. However, the effect of age (*χ*^2^ = 8.70, *df = *1, *p = *.003) and the interaction of *Sandwich Builder* scores and age (*χ*^2^ = 5.64, *df = *1, *p = *.02) both improved model fit. Older adults had poorer transcription accuracy than younger adults (*ß* = −0.07, *p = *.002), and the strength of the relationship between *Sandwich Builder* scores and performance increased with increasing age (*ß* = 0.01, *p = *.02). The interaction is visualized in Fig. 6 (left) using model fits of the bottom (18–30 years) versus top (52–65 years) quartiles of ages. The results using *Sandwich Builder* scores replicate those of McLaughlin et al. (2021), who measured memory ability using the Word Auditory Recognition and Recall Measure (WARRM; Smith et al., 2016): The relationship between short-term memory span and speech-in-noise transcription accuracy is more prominent in older participants. This finding aligns with prior work indicating a smaller role of short-term/working memory in supporting speech-in-noise comprehension in younger adults (Füllgrabe & Rosen, 2016). The full model is reported in Appendix D.

In the accent transcription task, log-likelihood model comparisons indicated that *Sandwich Builder* scores (*χ*^2^ = 22.26, *df = *1, *p < *.001) and age (*χ*^2^ = 5.89, *df = *1, *p = *.02) both significantly improved model fit. The interaction of *Sandwich Builder* scores and age was nonsignificant (*χ*^2^ = 2.91, *df = *1, *p = *.09); thus, the model without the interaction was taken as the full model (reported in Appendix D). Model estimates indicated that larger *Sandwich Builder* scores corresponded to better transcription accuracy (*ß* = 0.10, *p < *.001; Fig. 6, right). As in the speech-in-noise transcription task, older adults had poorer accent transcription accuracy than younger adults (*ß* = −0.01, *p = *.01).

#### Predictive power

Although the significant relationship between *Sandwich Builder* scores and performance in the speech-in-noise and accent transcription tasks demonstrated predictive validity, the question of predictive power remained. In other words, we sought to determine whether other researchers would need samples of *N = *209 (as in the current study) for *Sandwich Builder* to be an informative measure of individual differences or, rather, if *Sandwich Builder* could be valuable for research studies with smaller sample sizes as well.

To this aim, we simulated expected power curves using the current study’s speech-in-noise and accent transcription datasets. We focused on power to detect the main effect of *Sandwich Builder* score, i.e., the significance of *Sandwich Builder* score in a model predicting performance in the speech-in-noise task or (separately) in a model predicting performance in the accent transcription task. Although the interaction with age was significant in the speech-in-noise dataset, for simplicity we did not examine power to detect the interaction in this simulation.

**Fig. 7:**
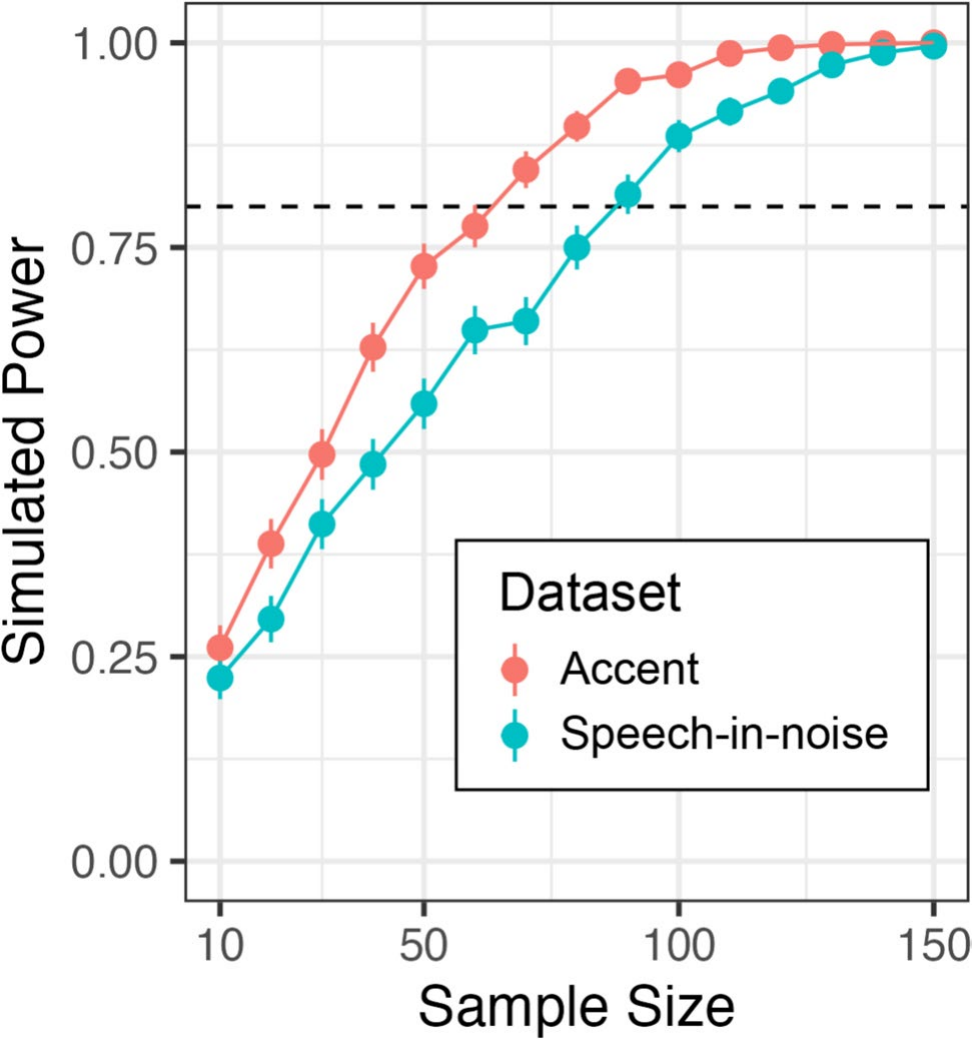
Simulated power at sample sizes spanning from 10 to 150 in steps of 10. Points represent mean power across simulated samples with 95% confidence interval bars. The dashed horizontal line denotes a power threshold of 0.80

At each sample size (from 10 to 150 in steps of 10), we conducted 1,000 tests on randomly sampled participants from our Session 2 dataset (Fig. 7). For example, this means that on each iteration of the simulation for the sample size of 10, we pulled data belonging to 10 participants at random from the pool of 209. Note that the trial set size was 16 for the speech-in-noise transcription dataset and 24 for the accent transcription dataset, which is a factor in addition to effect size that can impact expected power. The accent dataset reaches 80% power between 60 and 70 participants, and the noise dataset between 80 and 90 participants. Although these expected power estimates serve as a useful reference point, it is important to note that smaller or larger samples may be needed to reach sufficient power in future research. Power estimates are highly dependent on a task’s design/data type, model parameters, number of trials, and size of the expected effect. Datasets and simulation code are available for researchers who want to predict power for more complex effects (e.g., interactions), different trial set sizes, or different effect sizes. These kinds of manipulations on pre-existing data can be accomplished with R packages like *simr* (Green & MacLeod, 2016), making power simulations more informative for each given project.

### Discriminant validity

Discriminant validity was determined with Bayesian statistics in JASP (version 0.17.1; Love et al., 2019) using *Sandwich Builder* scores and the extraversion composite. As extroversion is not linked to memory span, these were expected to be unrelated (Waris et al., 2018). A default beta prior width of 1.0 was specified. Results indicated strong (classification from Quintana & Williams, 2018) evidence of a null relationship (*r = *0.05, *BF*_10_ = 0.11).

## Discussion

Although several cognitive tasks exist for assessing both visual and auditory short-term capacities in a laboratory environment (e.g., digit span), these types of tasks are typically not engaging for the participant—which may, in turn, negatively impact data quality (DeRight & Jorgensen, 2015). Experiment gamification is a growing strategy in cognitive research aimed at increasing participant motivation (Lumsden et al., 2016). By incorporating features common to videogames (e.g., graphics, competition, and/or narrative), the researcher can transform a boring experimental task into an engrossing game. In the current study, we assessed the efficacy of a novel gamified tool called *Sandwich Builder* for measuring auditory short-term memory capacity, while demonstrating the benefits of gamification on participant affect, fatigue, and motivation.

To establish the convergent validity of *Sandwich Builder*, we compared span scores obtained from a variety of short-term memory tasks. We adapted the classic digit span task from the Weschler Adult Intelligence Scale (Wechsler, 1987), using both an auditory and visual version; in these tasks, the participant is presented with sequences of numbers (of between 2 and 10 items) and then prompted to recall them. Following a similar design, we also created an auditory and visual version of a “free recall” task (modeled after Smith et al., 2016). The free recall task contains fewer trials, presenting 10 words (*market, college,* etc.) per trial and then prompting the participant to recall as many words as possible. A correlation analysis showed significant positive relationships between *Sandwich Builder* and all four of the other short-term memory tasks. The strength of these relationships varied: The strongest correlation was with the auditory free recall task, followed by the visual digit span task, auditory digit span task, and then the visual free recall task. In one way, this rank order is intuitive—the auditory free recall task (which presents words auditorily) is most similar to the *Sandwich Builder* task (which presents the sandwich ingredients auditorily). However, the auditory-modality tasks were not always more strongly correlated than the visual-modality tasks; the visual digit span showed the second-strongest correlation. Thus, the correlation analysis demonstrates the validity of *Sandwich Builder* as a measure of short-term memory—one that is relatively independent of modality.

Following each of the five (order-counterbalanced) short-term memory tasks, participants completed a questionnaire probing their affective state, level of fatigue, and motivation to perform well in the experiment. Participants exited the *Sandwich Builder* task in a better mood, less fatigued, and more motivated than they exited the other four memory tasks, reflecting the benefits of gamification. It is worth noting that these outcomes cannot be attributed to task duration; while *Sandwich Builder* takes approximately 9.5 minutes to complete, the free recall tasks take approximately 4.25 minutes, and the digit span tasks take approximately 9.25 minutes. In terms of number of trials, the free recall task is the shortest (*n = *6), followed by *Sandwich Builder* (*n = *12) and then digit span (*n = *36). The gamification in *Sandwich Builder* results in participants leaving the task happier, less fatigued, and more motivated, even when trials may be longer, and total time-within-task may be longer.

To assess test–retest reliability, participants were invited to return for a second session in which they completed *Sandwich Builder* again (approximately 2 weeks later). The intraclass correlation (ICC) of scores across sessions was 0.75, indicating moderate-to-good reliability (based on rule of thumb from Koo & Li, 2016). For research without clinical applications, we can conclude that *Sandwich Builder* has acceptable reliability (Nunnally, 1978). We also note that, given the same test combinations were repeated in each session (lending to greater practice effects), it may be the case that reliability in the current study was underestimated.

To determine the utility of *Sandwich Builder* as a measure of individual differences in speech perception and other cognitive research, we next examined predictive validity. Participants completed two speech transcription tasks previously shown to correlate with memory abilities (specifically, auditory working memory as estimated by the WARRM task; Smith et al., 2016). The first was a speech-in-noise transcription task based on McLaughlin et al. (2021), which found a relationship between accuracy and memory span in older adults. The second was a transcription task using L2-accented speech, based on McLaughlin et al. 2023), which found a relationship between accuracy and memory span in younger adults. For both tasks, our results replicated prior work—demonstrating that *Sandwich Builder* can capture the same individual differences in short-term memory abilities as the WARRM task.

By sampling a population ranging in age from 18 to 65, we also sought to address whether the relationship between *Sandwich Builder* scores and speech perception performance in these tasks may interact with participant age (i.e., whether relationships between *Sandwich Builder* scores and performance may be stronger in older than in younger adult populations). This additional aim was motivated by the fact that the relationship between accuracy and memory span found in McLaughlin et al. (2021) was only present in older—and not younger—adults; McLaughlin et al. (2023) only examined younger adults, so the question of whether older adults may be the same or different remained open. Our findings replicated McLaughlin et al. (2021): Performance during speech-in-noise perception was related overall to individual differences in short-term memory, with this trend driven by the older adult participants (i.e., there was a significant interaction with age). In the L2 accent transcription task, age did not significantly interact with individual differences in short-term memory. These outcomes align with prior speech perception research in younger adults showing a dissociation of the cognitive mechanisms supporting speech-in-noise processing versus L2 accent processing (McLaughlin et al., 2018). Although both types of listening conditions can present a challenge to inexperienced listeners, short-term and/or working memory appears to be more critical for processing the latter type of spoken language variation.

Results of our Bayesian analysis were as predicted, indicating strong evidence that *Sandwich Builder* scores have no relationship with a theoretically unrelated measure of personality (the measure of extraversion from the Big 5 Inventory; John & Srivastava, 1999), and therefore demonstrate discriminant validity. Thus, *Sandwich Builder* has the qualities typically desired of an individual differences measure: convergent validity, predictive validity, discriminant validity, and moderate-to-good test–retest reliability. The benefits that *Sandwich Builder* presents for the researcher over other classic tasks stem from its gamification (i.e., positive effects on mood, fatigue, and motivation).

In the present study, we examine *Sandwich Builder* as a measure of auditory short-term memory, given the “forward recall” nature of the task’s design. The nuance of what distinguishes a measure of short-term versus working memory, however, merits further discussion in this context. By definition, the distinction between short-term and working memory lies in the manipulation of held information: short-term memory refers to the cognitive system used for holding units of information before recall (e.g., digits or words) while working memory refers to the cognitive systems used for maintenance and manipulation before recall (Cowan et al., 2005). In practical research use, however, the same digit span tasks performed in forward (i.e., recalling 1, 2, 3 as 1, 2, 3) versus reverse (i.e., recalling 1, 2, 3 as 3, 2, 1) directions are typically referred to as a measures of short-term memory versus working memory, respectively—despite evidence to support this distinction remaining mixed (St Clair-Thompson, 2010). Indeed, a forward recall task like *Sandwich Builder* could be approached with multiple strategies; if a participant engages an acronym strategy (converting “ham, tomato, and pickles” to “H.T.P.” for easier recall), they will have, incidentally, engaged working memory. Thus, although the design of *Sandwich Builder* is most similar to a short-term memory task (like forward digit span), it could possibly also serve as a measure of working memory. Further, if the researcher wanted to intentionally increase engagement of working memory during the task, this could likely be accomplished via explicit instructions *(“Remember the ingredients by converting them to an acronym”*) or tweaking the task demands (e.g., changing *Sandwich Builder* into a reverse recall task).

Although *Sandwich Builder* incorporates multiple gamification elements (e.g., theme, levels, graphics, and animations), there are additional game-like features that could further increase positive effects on mood, fatigue, and motivation—for example, adding a progress bar at the top of the screen (showing the participant how far into the task they are), a scoring system (with a “top score” to try to beat), and/or positive (or negative) feedback. It also remains to be determined how each specific element (theme, levels, graphics, vs. animations) contributes to the cumulative benefits of gamification; for example, would the same outcomes be found if animations were removed from *Sandwich Builder*? Potential benefits to performance (on *Sandwich Builder* and/or surrounding experimental tasks) also remain to be investigated. Gamification has been found to positively impact participant performance in other settings (Ninaus et al., 2015), though whether this benefit is mediated by increased motivation remains to be seen. *Sandwich Builder* presents an excellent starting point for addressing these and other questions related to the benefits of experiment gamification.

The current design of *Sandwich Builder* is language-specific, including spoken and written language in English in the current version, with parameters chosen to be appropriate for adult participants. Alternative versions should be relatively easy to implement, given that the materials (including custom art) and *Sandwich Builder* experiment are openly available. For example, modifying *Sandwich Builder* for use in other languages would be valuable, and straightforward. There is also enormous potential for *Sandwich Builder* to benefit cognitive development research—indeed, experiment gamification could make vast improvements on data quality from child participants. Converting *Sandwich Builder* into a task well suited to ages with developing reading skills would require adapting the “sandwich-building scene,” for example, by including less text. As noted, because the code and materials for *Sandwich Builder* are openly accessible, any extension that a researcher might want is possible.

## Conclusion

Short-term memory is a critical cognitive mechanism that supports spoken language processing. Thus, reliably estimating individual differences in memory capacity is essential in psycholinguistics, as well as other cognitive science research. Classic tasks for estimating auditory short-term capacities (e.g., digit span) are typically not engaging for the participant and may lead to increased fatigue and/or poorer performance. An increasingly popular approach in psychological science for circumventing this issue is experiment gamification, which incorporates features common to videogames (e.g., graphics, competition, and/or narrative) into an experiment. In the current study, we examined a novel gamified tool, *Sandwich Builder*, for assessing individual differences in short-term memory capacity. *Sandwich Builder* shows strong convergent, discriminant, and predictive validity, and moderate-to-good test–retest reliability. Additionally, participants exit the *Sandwich Builder* task in a better mood, less fatigued, and more motivated (as compared to classic short-term memory tasks). We conclude that *Sandwich Builder* is a valid tool for assessment of short-term memory in linguistic and cognitive research of individual differences, with measurable advantages over other assessment tools. All materials (e.g., digital art) used in the creation of *Sandwich Builder*, as well as the ready-to-run *Sandwich Builder* experiment (built with Gorilla’s Game Builder; Anwyl-Irvine et al., 2020), are openly available for use by other researchers.

## Supplementary Information

Below is the link to the electronic supplementary material.

Supplementary file1 (DOCX 437 KB)

## Data Availability

The datasets generated and analyzed during the current study are available in the Open Science Framework repository: https://osf.io/snmqt/files/osfstorage.

## Footnotes

1 We make a minor deviation from our pre-registration in outlier exclusions. We had pre-registered that we would exclude participants more than three mean absolute deviations (MADs) from the mean, but opted for a stricter threshold (five MADs) based on visual inspection of the data.

2 Experiment gamification requires trade-offs in experimental control and task engagement. Varying syllable lengths of the sandwich ingredients may increase the variation of within-level trial difficulty; for example, a Level 2 trial with “arugula and cucumber” may differ in challenge from a Level 2 trial with “egg and ham.” Similarly, ingredients like “tofu” and “arugula” are not common, but they each add to the aesthetics and visual diversity of *Sandwich Builder,* and thus the quality of gamification. For other research purposes, *Sandwich Builder* is designed to be easy to modify to allow stimulus sets with fixed lengths, if desired.

3 The instructions for the free recall task encouraged participants to recall items in order; however, the scoring procedures credited disordered sequences. Thus, we refer to the task as “free recall” but note that from the participant point of view, it was similar to a “forward recall” task.

4 There are benefits but also potential drawbacks to the adaptive procedure chosen for *Sandwich Builder.* This procedure (as described and implemented in the current study) requires fewer trials, thus reducing the overall task length. However, for some special populations it may be beneficial to use a more traditional design in which the trial difficulty gradually increases—even at the cost of a longer task. This change in procedure can be accomplished by setting the initial spreadsheet for the *Sandwich Builder* module (within an experiment flowchart in Gorilla’s Design tab) to Level 1 instead of Level 6.

5 The scoring procedure for *Sandwich Builder* was developed using the Session 1 data before the results were known. The procedure described in this section (i.e., summing “base” and “partial credit” scores) was ultimately chosen because it captured participants’ maximal capabilities with only minor “partial credit” adjustments after the fact. For example, another scoring procedure that was considered during this exploratory process instead identified the modal (most frequent) level passed as the participant’s span capacity; however, visual inspection of the trial-to-trial data indicated that this scoring option did not accurately capture participants’ maximal capabilities, and thus would have required a more complex “partial credit” procedure. Although multiple procedures were explored in this phase, the effect of each procedure on the statistics of interest (e.g., correlations tests) were not compared, so as to prevent researcher bias. Only population-wide distributions were examined during this stage with the aim of confirming a relatively normal distribution of scores.

6 An effect of hearing ability, as estimated by the Hearing Handicap Inventory for the Elderly (HHIE) questionnaire, was examined but ultimately dropped as it did not improve model fit in any instances (*p*s >.05).

## Code availability

The code used for stimuli generation and analysis in the current study are available in the Open Science Framework repository: https://osf.io/snmqt/files/osfstorage.
