# Supplementary material for: Lettuce entertain you: Assessing Sandwich Builder as a measure of auditory short-term memory
Source: Behav Res Methods. 2025 Jun 4;57(7):191. doi: 10.3758/s13428-025-02707-1 (PMC12137375; doi:10.3758/s13428-025-02707-1)
Supplement: Supplementary file 1 — Supplementary file1 (DOCX 437 KB) [file 13428_2025_2707_MOESM1_ESM.docx]

**Appendix A**

List of 21 Sandwich Ingredients and Images


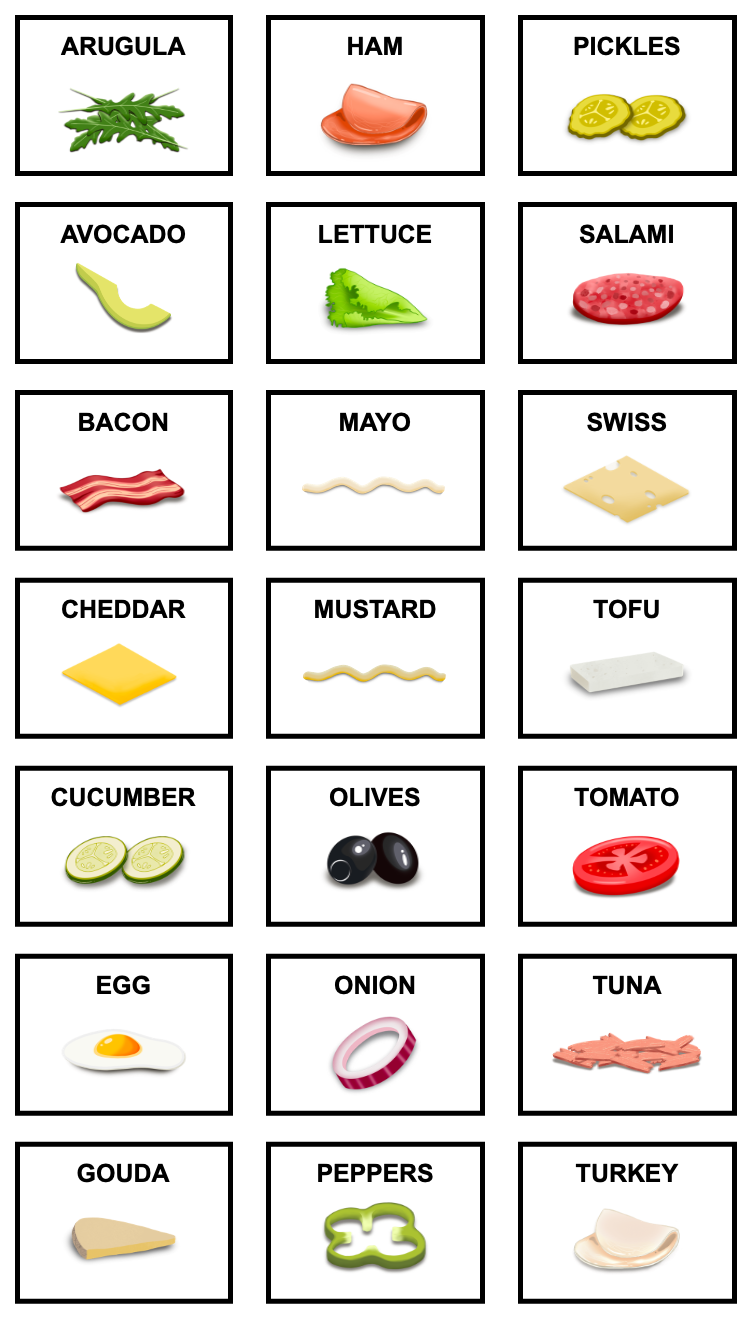


**Appendix B**

List of Post-Task Questions (Affect, Fatigue, and Motivation)

Affect:

1. How happy do you feel right now?
2. How interested do you feel right now?
3. How enthusiastic do you feel right now?
4. How annoyed do you feel right now?
5. How irritated do you feel right now?
6. How frustrated do you feel right now?

Fatigue:

1. How tired do you feel right now?
2. How energetic do you feel right now?

Motivation:

1. How motivated were you to perform well in the task you just completed?
2. How much did you desire to perform well in the task you just completed?

**Appendix C**

*R Model Syntax*

**Speech-in-Noise Transcription: Full Model**

glmer(cbind(Correct, Incorrect) ~ 1 + SB_score + Age + SB_score:Age +

(1 | Subject) + (1 | Audio),

data = noise_data, family = "binomial",

control = glmer_control_settings)

**Accent Transcription: Full Model**

glmer(cbind(Correct, Incorrect) ~ 1 + SB_score + Age +

(1 | Subject) + (1 | Audio),

data = accent_data, family = "binomial",

control = glmer_control_settings)

**Model Control Statement:**

glmer_control_settings <- glmerControl(optimizer = "bobyqa",calc.derivs=FALSE,

optCtrl=list(maxfun=1e9),

check.conv.grad = .makeCC("warning", tol = 1e-3,

relTol = NULL),

check.conv.singular = .makeCC(action = "message",

tol = 1e-9),

check.conv.hess = .makeCC(action = "warning", tol = 1e-6))

**Appendix D**

*Full Model Summaries*

**Table D1**

*Speech-in-Noise Transcription: Full Model*

| **Fixed Effects** | |  |  |  | |  | |
| --- | --- | --- | --- | --- | --- | --- | --- |
| **Predictor** | | **Coefficient (𝛽)** | **S.E.** | ***Z*** | | ***p*** | |
| Intercept | | 2.37 | 1.09 | 2.18 | | .03 | |
| *Sandwich Builder* score | | -0.19 | 0.20 | -0.97 | | .33 | |
| Age | | -0.07 | 0.02 | -3.15 | | .002 | |
| *Sandwich Builder* score : Age | | 0.01 | < 0.01 | 2.39 | | .02 | |
| **Random Effects** | |  |  |  | |  | |
| **Predictor** |  | **Variance** | **S.D.** |  | | | |
| Subject | (Intercept) | 1.46 | 1.21 |  |  |  |  |
| Item | (Intercept) | 0.52 | 0.72 |  | |  | |

**Table D2**

*Accent Transcription: Full Model*

| **Fixed Effects** | |  |  |  | |  | |
| --- | --- | --- | --- | --- | --- | --- | --- |
| **Predictor** | | **Coefficient (𝛽)** | **S.E.** | ***Z*** | | ***p*** | |
| Intercept | | -0.22 | 0.20 | -1.13 | | .26 | |
| *Sandwich Builder* score | | 0.10 | 0.02 | 4.84 | | < .001 | |
| Age | | -0.01 | < 0.01 | -2.45 | | .01 | |
| **Random Effects** | |  |  |  | |  | |
| **Predictor** |  | **Variance** | **S.D.** |  | | | |
| Subject | (Intercept) | 0.13 | 0.36 |  |  |  |  |
| Item | (Intercept) | 0.36 | 0.60 |  | |  | |

**Supplemental Materials**

**Sandwich Builder Demo**

YouTube Link: <https://youtu.be/ZVY6AkHlKx0>

**Figure S1**

*Outlier Identification*

*
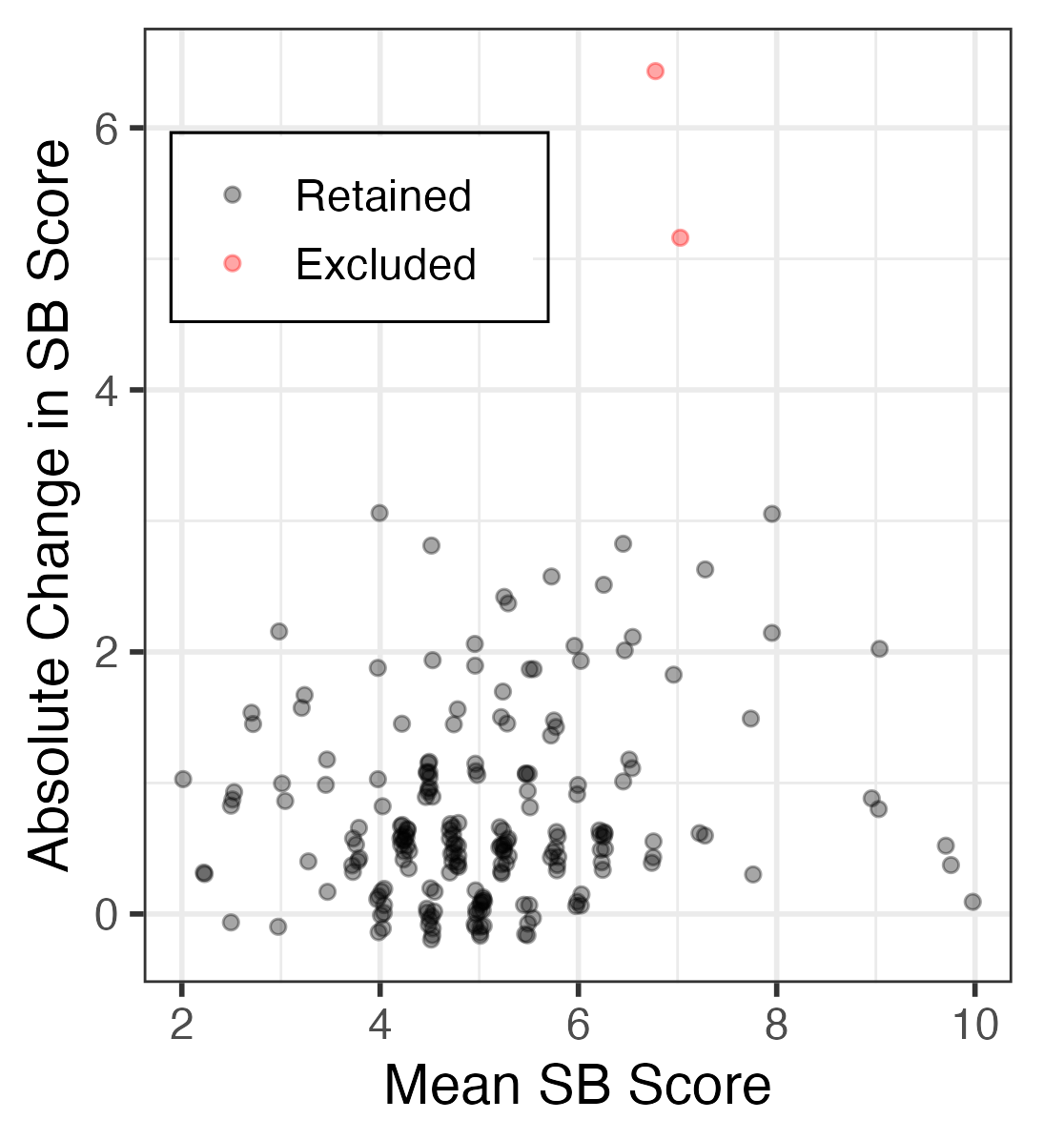
*

*Note.* Outliers (*n* = 2) were identified based on absolute change in *Sandwich Builder* (SB) scores from Session 1 to Session 2. All participants are shown with points based on their mean SB score (x-axis, jittered for visualization purposes) and absolute change in SB score. Excluded participants had extreme changes from Session 1 to Session 2 (more than five mean absolute deviations from the mean).
